# Topoisomerase II isoforms are required for HIV-1 reverse transcription

**DOI:** 10.1186/1471-2334-12-S1-P75

**Published:** 2012-05-04

**Authors:** Lokeswara Balakrishna Sunnam, N Satyanarayana, Anand K Kondapi

**Affiliations:** 1 School of Life Sciences, University of Hyderabad, Hyderabad, India

## Background

Human topoisomerase II is present in two forms 170kDa α and 180 kDa β isoform. Early reports have indicated that the topoII poisons and antisense RNA can block viral replication. The objective of present study is to evaluate specific requirement of topoII isomerases in promoting HIV replication and to characterize the HIV-1 replication intermediates to study molecular action of the topoII α or/and β isoforms.

## Methods

In topoII α or/and β were down regulated by using siRNA mediated gene silencing in SupT1 cells and infected with HIV-1 (93IN101) at mentioned time points DNA and RNA were isolated and analyzed by PCR. Co-localization studies were done by using fluorescent antibodies specific to RT, topoII α and topoII β and images were taken in confocal microscopy.

## Results

The results indicated that HIV-1 replication was aborted in topoII α or/and β down regulated SupT1 cells. Analysis of the intermediates formed during the HIV-1 replication cycle in topoII α-, topoII β- and topoII α- β- SupT1 cells do not support viral gene expression, integration, PICs formation and cDNA synthesis. Moreover, results revealed the topoII α and β co-localization with HIV-1 reverse transcriptase. Taken together, results display the requirement of topoisomerase II isoforms in the event of HIV-1 reverse transcription.

## Conclusion

Topoisomerase II isoforms are involved in the HIV-1 lifecycle in the early event of HIV-1 reverse transcription, influencing the phenomenon through an unknown mechanism.

